# Effects of Obesity on Medial Tibiofemoral Cartilage Mechanics in Females—An Exploration Using Musculoskeletal Simulation and Probabilistic Cartilage Failure Modelling

**DOI:** 10.3390/life13020270

**Published:** 2023-01-18

**Authors:** Jonathan Sinclair, Holly Lynch, Nachiappan Chockalingam, Paul John Taylor

**Affiliations:** 1 School of Sport & Health Sciences, Faculty of Allied Health & Wellbeing, University of Central Lancashire, Preston PR1 2HE, UK; 2 School of Life Sciences and Education, Staffordshire University, Stoke on Trent ST4 2DE, UK; 3 School of Psychology & Computer Sciences, Faculty of Science & Technology, University of Central Lancashire, Preston PR1 2HE, UK

**Keywords:** biomechanics, obesity, osteoarthritis, cartilage, musculoskeletal simulation, probabilistic modelling

## Abstract

This study examined the effects of obesity on cartilage mechanics and longitudinal failure probability at the medial tibiofemoral compartment, using combined musculoskeletal simulation and probabilistic failure modelling approaches. The current investigation examined twenty obese females (BMI > 30.0 kg/m^2^) and 20 healthy weight (BMI < 25.0 kg/m^2^) females. Walking kinematics were obtained via an 8-camera optoelectric system, and a force plate was used to collect ground reaction forces. Musculoskeletal simulation and probabilistic failure modelling were utilized to explore medial tibiofemoral forces and cartilage probability. Comparisons between groups were undertaken using linear mixed-effects models. Net peak cartilage forces, stress and strain were significantly larger in the obese group (force = 2013.92 N, stress = 3.03 MPa & strain = 0.25), compared to health weight (force = 1493.21 N, stress 2.26 MPa & strain = 0.19). In addition, medial tibiofemoral cartilage failure probability was also significantly larger in the obese group (42.98%) compared to healthy weight (11.63%). The findings from the current investigation show that obesity has a profoundly negative influence on longitudinal medial knee cartilage health and strongly advocates for the implementation of effective weight management programs into long-term musculoskeletal management strategies.

## 1. Introduction

Obesity is present in over a third of the adult population in the United States [1] and continues to escalate at an alarming rate [2]. Increased adiposity is linked to the aetiology of heart disease, diabetes, stroke and many forms of cancer [3] and also regarded as the fifth leading risk factor for mortality, causing over 2.8 million deaths annually [4]. In addition, obesity also increases the risk for comorbidities, including chronic musculoskeletal pathologies [5]. Therefore, the rapidly accelerating rates of global obesity raise concerns regarding associated increases in the prevalence of musculoskeletal disorders.

Osteoarthritis (OA) is the most frequently experienced chronic musculoskeletal pathology and represents the foremost cause of enduring disability among older adults [6]. Tibiofemoral OA the most frequently experienced form of OA, is the principal cause of international musculoskeletal disability [7] and has a negative fiscal influence on global healthcare systems [8]. Knee OA is representative of a degenerative articular cartilage disease, illustrated by deterioration of the cartilage itself within the knee joint [9]. Affected individuals importantly experience enduring pain and dysfunction [10], and knee joint OA has been shown have an incidence rate of almost 10% in individuals aged 60 and above [11]. Knee OA cases are most frequent in females and predominantly detected at the medial compartment of the tibiofemoral joint, as loads borne by this joint predominantly pass through the medial aspect of the knee [12].

Longitudinal analyses have shown that obesity may be a significant risk factor for the instigation and advancement of symptomatic and radiographic tibiofemoral OA, but have not conclusively exposed the mechanisms responsible for this link [13]. Multiple hypotheses have been advocated that expound the association between obesity and chronic musculoskeletal disorders [14]. Mechanically it is advocated that excessive axial loading mediated through increased body mass in obese individuals, promotes degeneration of lower extremity joint structures [15]. However, whilst mechanical inferences predominate, metabolic factors associated with obesity including hormonal and biochemical alterations have also been proposed as being responsible for the incidence of musculoskeletal disorders in obese individuals [16]. Furthermore, accumulating evidence also shows that obesity mediates systemic low-grade inflammation, that contributes to metabolic dysfunction [17]. Adipose tissue itself is functional as an active endocrine organ by releasing bioactive substances known adipokines, which are able to mediate either pro or anti-inflammatory activities [18]. There has been considerable research interest concerning the potential role of adipokines in the pathogenesis of OA [19]. Previous analyses have confirmed that levels of leptin and resistin were greater in obese individuals with knee OA in relation to healthy controls and that levels of these adipokines were also associated with radiographic OA stage [20], indicating that they may play a significant role in the multifactorial nature of knee OA pathophysiology [21].

Currently, the implications of obesity during everyday tasks such as walking, remain poorly understood. Obese individuals exhibit a reduced self-selected walking velocity [22,23]; alongside shorter absolute stride and step lengths [22,24]. Kinematically, obese individuals have been shown to adopt a significantly increased hip adduction angle [24], hip extension angle [25] and peak hip extension moment [26]. At the knee joint, obese individuals exhibit reduced knee flexion at initial contact [26], peak flexion [25], and an enhanced knee extension moment [26,27]. In the coronal plane, obese individuals are associated with a greater knee adduction moment (KAM) during weight acceptance [28], peak KAM [26] and KAM impulse [27]. At the ankle joint, obese individuals are associated with increased dorsiflexion throughout the stance phase [24], greater inversion at footstrike [29], an increased peak eversion angle [23] and increased toe-out throughout the stance phase [22,29,30]. In addition, obese individuals also exhibited a statistically greater ankle plantarflexion moment [25,26].

However, joint moments are not characteristic of localized joint loading [31], and it is the tibiofemoral joint contact forces that are linked to the initiation and progression of cartilage breakdown. Considerable advances in musculoskeletal simulation modelling have been made [32], allowing skeletal muscle driven indices of lower extremity joint reaction forces to be calculated [12]. Lerner et al. [33] examined the effects of paediatric obesity on tibiofemoral joint compressive loading and showed that obese children had significantly greater compressive forces and that body mass index (BMI) predicted the percentage of total tibiofemoral load borne by the medial compartment. Harding et al. [34] examined the effects of overweight (BMI > 25.0) and obese (BMI > 30.0) individuals on muscle and medial tibiofemoral compartment forces compared to healthy weight (BMI < 25.0) participants. Their findings showed that in relation to healthy weight participants, obese individuals exhibited greater quadriceps and medial tibiofemoral compartment forces. 

However, although quantification of tibiofemoral joint kinetics is now feasible, there remain difficulties in exploring the influence of distinct mechanical and physiological conditions on the instigation, progression and temporal profile of knee OA. Therefore, probabilistic modelling of cartilage of stress and strain induced accumulative damage may be valuable for determining the effects of obesity on probability of osteoarthritic degeneration over a lifetime of cyclic loading [35]. However, probabilistic cartilage modelling has not been adopted to explore differences in medial tibiofemoral cartilage failure probability between obese and healthy weight individuals.

The aim of the current investigation was to explore the effects of obesity on medial tibiofemoral cartilage mechanics and lifetime failure probability in relation to healthy weight individuals using a combined musculoskeletal simulation and computational modelling approach. The findings from this investigation will yield new information firstly on the effects of obesity on medial tibiofemoral cartilage mechanics during walking, but also on lifetime failure probability in relation to healthy individuals. This study tests the hypothesis that medial-tibiofemoral cartilage loading mechanics and lifetime failure probability will be significantly greater in the obese group compared to healthy individuals.

## 2. Materials and Methods

### 2.1. Participants

Twenty healthy weight and twenty obese female participants volunteered to take part in this study. Participants not eligible for this study if they had a current lower extremity pathology or had previously undergone lower extremity surgery. Body mass index (BMI) was utilized to define obesity (BMI > 30.00 kg/m^2^) and healthy weight groups (BMI < 25.00 kg/m^2^) due to its adoption in clinical practice, as well as its correlation with more accurate measures of adiposity [36]. Using data from previous work in obese and healthy weight adults [34] and mean ± SD values for the peak net medial tibiofemoral force of 1227.30 ± 521.30 N in healthy weight and 1812.90 ± 703.80 N in obese individuals, it was determined using GPower software (GPower 3.1) that for between group comparisons, to achieve *α* = 5% and *β* = 80%, that 40 total participants would be required. All participants provided consent in written form in accordance with the ideologies outlined in the Declaration of Helsinki. The methodological approach adopted in the current study was approved by an institutional ethics panel (STEMH 1013).

**Figure 1:**
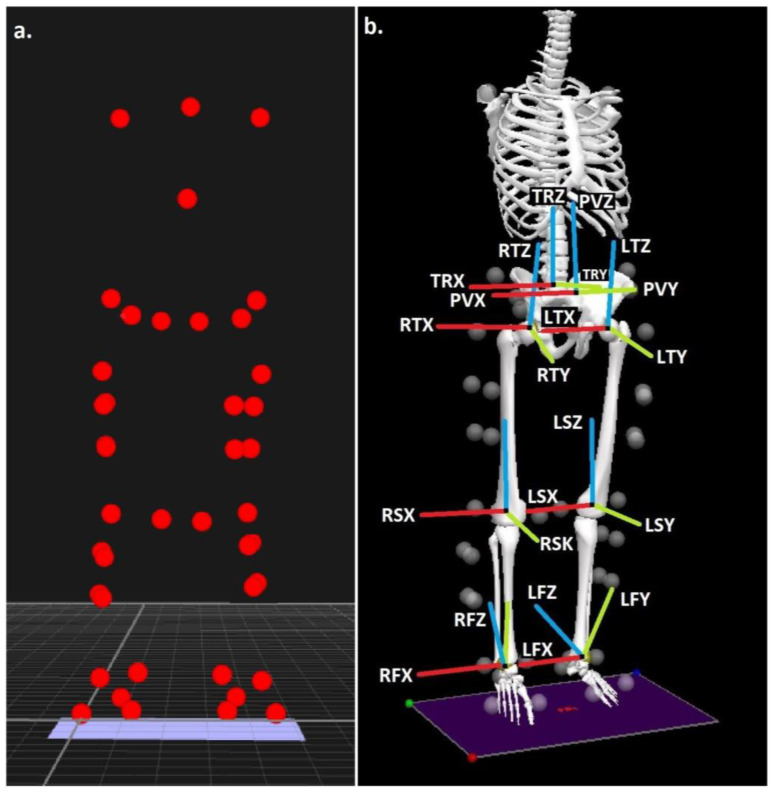
(**a**) Anatomical landmark locations and (**b**) modelled segments, with segment co-ordinate axes (R = right & L = left), (TR = trunk, P = pelvis, T = thigh, S = shank & F = foot), (X = sagittal, Y = coronal & Z = transverse planes).

Body segments were modelled in 6 degrees of freedom using the calibrated anatomical system technique [37], using a marker/ model configuration utilized previously to quantify the biomechanics of walking [12]. (Figure 1). Intra rater reliability for the individual responsible for positioning of the anatomical markers has been shown to be high (ICC ≥ 0.931) [38]. The centres of the ankle and knee joints were the midpoints between the malleoli and the femoral epicondyle markers [39,40] and the hip joint centre was established via a regression approach using the locations of the anterior superior iliac spine markers [41].

### 2.2. Procedure

Retroreflective marker data were obtained using an 8-camera optoelectric motion capture system (Qualisys Medical, Gothenburg, Sweden) operating at 250 Hz. Dynamic calibration of the camera system was undertaken prior to each testing session. A piezoelectric force plate (Kistler Instruments, Winterthur, Switzerland) operating at 1000 Hz was utilized to capture ground reaction forces (GRF). Kinematic and GRF data were collected in a synchronous manner. 

The calibrated anatomical system technique (CAST) [37], was adopted to reconstruct body segments in 6 degrees of freedom. A marker/ modelling configuration that has been previously utilized to quantify walking biomechanics was adopted [12] (Figure 1). Intra rater reliability for the individual in control of placing the anatomical markers has been shown through previous publication to be very high (ICC ≥ 0.931) [38]. The ankle and knee joint centres were located at the centre point of the malleoli and the femoral epicondyle markers [39,40] and centre of the hip joint was ascertained using the locations of the anterior superior iliac spine markers via a regression based approach [41].

### 2.3. Processing

Digitization of the dynamic walking trials was undertaken using Qualisys Track Manager (QTM) (Qualisys Medical AB, Gothenburg, Sweden) software. Digitized QTM files were then exported in.C3D file format into Visual 3D (C-Motion, Germantown, MD, USA). Data were time normalized within Visual 3D to identify the stance phase, which was defined as the period over which the force plate measured > 20 N of vertical GRF [42]. Three-dimensional marker trajectories and GRF’s were smoothed with cut-off frequencies of 6 and 50 Hz, respectively, via a 4th-order low-pass zero-lag Butterworth filter. Cut-off frequencies were optimized using residual analysis for both kinetic and kinematics data [43]. The velocity of walking (m/s) was calculated as mean linear velocity of the model centre of mass in the anterior direction during the stance phase using Visual 3D [44]. Stride length (m) was determined as the linear anterior distance in the foot centre of mass location at footstrike between initial and subsequent ipsilateral footfalls [45].

#### 2.3.1. Medial Tibiofemoral Forces

Walking data during the stance phase were exported into bespoke musculoskeletal simulation software (OpenSim v3.3, Simtk.org). A validated musculoskeletal model [46] was firstly scaled to account for the anthropometrics of each participant. Dynamic inconsistency was solved using a residual reduction algorithm function within OpenSim [32]. Muscle kinetics were then quantified using a weighted static optimization process [47]. A joint reaction analysis process within OpenSim was then utilized, using muscle force data generated via static optimization [32]. The peak net (N) and normalized medial tibiofemoral forces (BW) were calculated using data derived from the joint reaction analysis. The cumulative medial tibiofemoral load was computed as the quotient of the mean stance phase medial tibiofemoral force and the stride length [35]. Pilot walking data shows a minimal detectable difference (MDC) of 0.27 BW and a high level of reliability (ICC = 0.951) for the peak medial tibiofemoral force (Table S1). Sinclair et al. [45] also importantly showed that vastus intermedius, vastus lateralis and vastus medialis muscle forces at the instance of peak joint force, were the strongest predictors of peak medial tibiofemoral joint loading during walking. Therefore, to determine the mechanisms responsible for any alterations in peak medial tibiofemoral kinetics between footwear conditions, the peak net (N) and normalized forces (BW) quantified during static optimization for the aforementioned muscles were quantified at the instance of peak joint force and extracted for statistical analysis.

#### 2.3.2. Medial Tibiofemoral Contact Mechanics

The medial tibiofemoral contact forces obtained from OpenSim were input into a model of medial knee contact mechanics which was utilized to calculate tibiofemoral cartilage stress and strain via adapted MATLAB source code [35]. Pilot walking data shown MDCs of 0.01 and 0.16 MPa and high reliability (ICC ≥ 0.945) for indices of medial tibiofemoral stress and strain (Table S1). The tibiofemoral contact model is based on that outlined by Nuño and Ahmed [48]. Medial tibiofemoral stresses (σ) and strains (ε) were quantified using the formulae outlined in Equations (1) and (2).

EQ1: σ = − Mean tibiofemoral cartilage modulus ∗ (Log (1 − ε))(1)

EQ2: ε = Cartilage element compression − modelled cartilage height(2)

The medial femoral condyle was modelled from a sagittal viewpoint as two convex curves denoting its anterior and posterior components, and from a coronal posterior perspective as a single arc. Conversely, the tibial plateau was denoted as a concave arc. The radii of the anterior and posterior components of the femoral arc in the sagittal plane were 35.0 mm and 18.9 mm, whereas that of the tibial arc in the frontal plane was modelled as 21 mm. In accordance with the Nuno & Ahmed [48] model, the tibia was considered to-be held in a fixed position-in space and the femur featured two modifiable components: the-axial height of the knee flexion axis relative to the tibia and the knee flexion angle itself. The tibiofemoral joint cartilage itself was modelled as a series of elements on the tibial plateau, with an unloaded height of 5.0 mm [49]. The cartilage elements were assumed to display a nonlinear elastic stress-strain relationship [50].

Contact stress (MPa) and strain at the medial tibiofemoral compartment were obtained using modelled indices for cartilage moduli, compression magnitude of the modelled cartilage contact elements as well as the quantity of contact elements. The modelled contact elements were 7326, which reflected a distance of 0.5 mm between elements, and the aforementioned tibiofemoral radii. The moduli of the cartilage included in the model were distinct for the different locations as some were covered by the medial meniscus. The femoral cartilage, unconcealed tibial cartilage and concealed tibial cartilage, were considered to have moduli values of 8.6, 4.0 and 10.1 MPa [51]. The medial meniscus modulus was included into the model as 1.3 MPa, and the meniscus itself considered to obscure 46 % of the tibial plateau [52,53]. The cartilage and menisci elements were described with a modelled Poisson’s ratio of 0.45 [54].

The angle of knee flexion was included into the contact model as that at which the peak medial tibiofemoral contact force (obtained using musculoskeletal simulation) occurred. The axial elevation of the knee flexion axis was incrementally decreased until the modelled peak medial tibiofemoral contact force matched that provided from musculoskeletal simulation. As the modelled tibiofemoral radii are distinct, the location of the loaded articular cartilage also differed alongside changes in the angle of knee flexion [55]. As the medial femoral condyle has been shown to remain close to the centre of the tibial plateau with alterations in the angle of knee flexion, it was determined that inclusion of translational knee joint mechanics were not necessary for this model [56].

#### 2.3.3. Medial Tibiofemoral Cartilage Failure Probabilistic Modelling

Medial tibiofemoral cartilage failure was defined as macroscopical plastic deformation typically observed in early-stage OA cartilage deterioration [57]. As 55 years has been demonstrated as the median age for knee OA diagnosis, and 9.29% of the US population is diagnosed with symptomatic knee OA by age 60 [11]; probability of cartilage failure was quantified across a duration of 42 years, from anatomical musculoskeletal maturity aged 18 until 60 years of age [58]. Probability of cartilage failure was quantified using probabilistic modelling; incorporating indices of both damage and repair [59,60,61], with cartilage strain the primary input parameter for damage. Sensitivity analyses were undertaken to determine the sensitivity of the key cartilage failure determinant, i.e., peak tibiofemoral strain to alterations (within feasible biological/anthropometric ranges) of each modelled parameter separately whilst maintaining the others at their modelled values (Tables S2 and S3 and Figures S1–S8).

Cartilage failure probability across the quantified duration, was obtained as a collective function of the aforementioned articular cartilage properties experiencing loading cycles over the daily modelled distance, using the stride length from the initial processing section to determine the daily number of loading cycles using Equation (3).

EQ3: Probability of cartilage failure = 1 − Exp − [(Volume of stressed cartilage / Reference stressed cartilage volume)(time − time until failure)Weibull exponent /Power law exponent](3)

In Equation (3), constants in the probabilistic cartilage failure model were the reference cartilage volume (78.5 mm^3^), Weibull exponent (14.3) and power law exponent (12.9). Time until failure of the articular cartilage was quantified using Equation (4).

EQ4: Time to failure = (Power law coefficient ∗ Stride length / Distance-per day) (Weibull coefficient ∗ ε) − Power law exponent (4)

In Equation (4), the time until failure is representative of the duration at which 63.2% of cases would experience failure after undergoing the magnitude and volume of cartilage strains. Daily distance travelled was included in the model as 6.0 km, which represents the approximate distance covered, had 7000 steps (the number now considered optimal for health and wellbeing [62] been completed per day, taking into account the stride lengths obtained from the current investigation. The power law coefficient (1.0), Weibull coefficient (1.03) and power law exponent (12.9) were incorporated as constants in the quantification of time until failure. These parameters were extracted from Miller & Krupenevich, [35], who fit a power law function to the loading cycles to failure data of Riemenschneider et al. [63].

Equation (3) shows in vitro failure probability. As living cartilage does possess limited innate ability to recover from strain-induced damage over time [64], the probability of medial tibiofemoral cartilage repair included into the failure model using Equation (5).

EQ5: Probability of repair = 1 − Exp − [ − (time / time until repair) Cartilage repair exponent] (5)

In Equation (5), the cartilage repair exponent (5.2) and time until repair (5.0 years) were modelled as constants [35], and the repair duration was correspondingly included as the time after which repair would be anticipated in 63.2% of cases of damage.

A probability density function determining the instantaneous probability of failure at a given time, was utilized to encompass repair into Equation 3 [35]. This is delineated in Equation (6).

EQ6: Probability density function = (Volume of stressed cartilage ∗ Weibull exponent / Power law-exponent ∗ Reference stressed cartilage volume ∗ time until failure) (time / time until failure) Weibull exponent / Power law exponent − 1 Exp [− (Volume of stressed cartilage / Reference stressed cartilage volume) (time / time until failure) Weibull exponent / Power law exponent](6)

The product of the probability density function and the communal probability that repair had not yet occurred, was integrated as a function of time in order to ascertain failure probability with repair. This procedure is described in Equation 7.

EQ7: Probability of failure with repair = ∫ (time 0) [Probability density function ∗ (1 − Probability of repair)] Modelled distance between contact elements ∗ time (7)

### 2.4. Statistical Analyses

For each biomechanical and cartilage failure outcome variable; means, standard deviations (SD) and 95% confidence intervals (95% CI) around the mean were calculated. To compare participant characteristics, biomechanical and cartilage failure outcomes between healthy weight and obese groups, between groups linear mixed effects models were adopted using the restricted maximum-likelihood method, with group (i.e., obese/healthy weight) included as a fixed factor and random intercepts modelled by participants [44]. Linear regression analysis was also adopted in both groups to determine the relationship between BMI and peak force, peak stress and peak strain. All statistical analyses were conducted using SPSS v27 (IBM, SPSS). For linear mixed models, the mean difference (*b*), t-value, and 95% CI of the difference are presented. Statistical significance for all analyses was accepted at the *p* < 0.05 level.

## 3. Results

### 3.1. Participant Characteristics

There were no differences in between groups for age (*b* = 1.32 _(95% CI = −1.07–3.70)_, t = 1.12, *p* = 0.27) and stature (*b* = 0.00 _(95% CI = −0.03–0.03)_, t = 0.03, *p* = 0.98) between groups. However, both body mass (*b* = 29.64 _(95% CI = 26.78–32.49)_, t = 20.97, *p* < 0.001) and BMI (*b* = 11.16 _(95% CI = 10.01–12.31)_, t = 19.54, *p* < 0.001) were significantly greater in the obese group (Table 1).

### 3.2. Initial Kinematic Processing

No significant differences in walking velocity were found between the two groups (*b* = 0.07 _(95% CI = −0.07–0.21)_, t = 0.98, *p* = 0.33). In addition, there were no significant differences between groups for stride length was (*b* = 0.06 _(95% CI = −0.06–0.19)_, t = 0.99, *p* = 0.31) (Table 2).

### 3.3. Medial Tibiofemoral Forces and Muscle Forces

There were no differences in peak normalized medial tibiofemoral force (*b* = 0.00 _(95% CI = −0.31–0.32)_, t = 0.02, *p* = 0.98) or normalized cumulative load (*b* = 0.14 _(95% CI = −0.07–0.34)_, t = 1.32, *p* = 0.19) between groups. However, peak net medial tibiofemoral force (*b* = 520.71 _(95% CI = 212.70–828.72)_, t = 3.42, *p* < 0.001) and net medial tibiofemoral cumulative load were shown to be significantly greater in the obese group (*b* = 452.19 _(95% CI = 270.36–634.02)_, t = 5.03, *p* < 0.001) (Table 3).

There were no differences in normalized vastus intermedius (*b* = 0.00 _(95% CI = −0.31–0.32)_, t = 0.02, *p* = 0.98), vastus lateralis (*b* = 0.00 _(95% CI = −0.31–0.32)_, t = 0.02, *p* = 0.98) or vastus medialis (*b* = 0.00 _(95% CI = −0.31–0.32)_, t = 0.02, *p* = 0.98) forces. However, net vastus intermedius (*b* = 153.23 _(95% CI = 7.26–299.19)_, t = 2.17, *p* = 0.04), vastus lateralis (*b* = 134.69 _(95% CI = 6.39–262.99)_, t = 2.13, *p* = 0.04) and vastus medialis (*b* = 113.82 _(95% CI = 4.91–222.74)_, t = 2.12, *p* = 0.04) forces were significantly greater in the obese group (Table 3).

### 3.4. Medial Tibiofemoral Contact Mechanics

Peak medial tibiofemoral stress was significantly greater in the obese group (*b* = 0.77 _(95% CI = 0.23–1.31)_, t = 2.90, *p* = 0.01), Furthermore, peak tibiofemoral strain was also found to be significantly greater in the obese group (*b* = 0.06 _(95% CI = 0.02–0.09)_, t = 2.97, *p* = 0.01) (Table 4).

### 3.5. Medial Tibiofemoral Cartilage Failure Probabilistic Modelling

**Figure 2:**
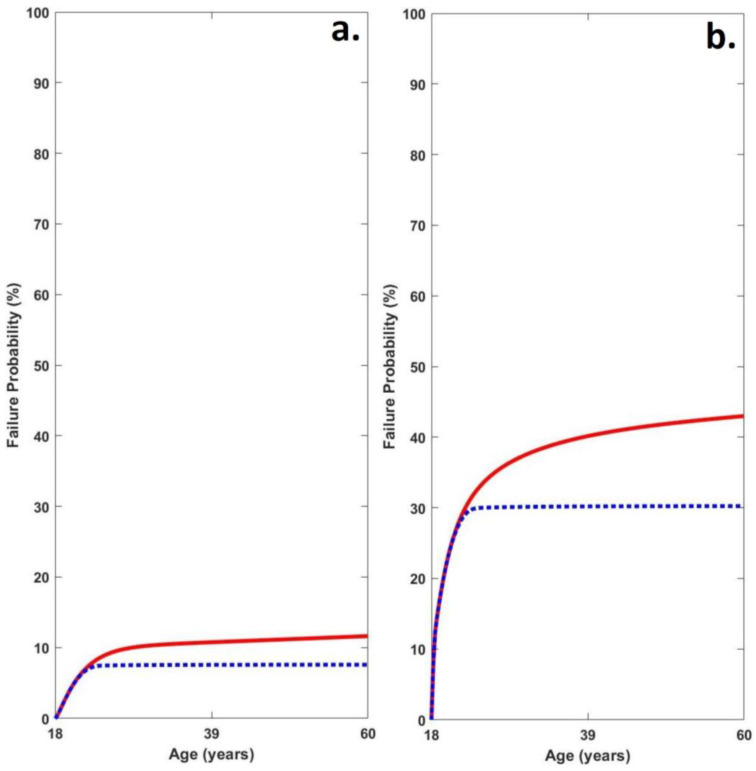
Average medial tibiofemoral cartilage failure time series probabilities in (**a**) healthy weight and (**b**) obese groups (red line = failure probability without adaptation and blue line = failure probability with adaptation).

Probability of failure was significantly greater in the obese group (*b* = 31.35 _(95% CI = 5.81–56.90)_, t = 2.48, *p* = 0.02). Furthermore, Probability of failure with repair was also found to be significantly greater in the obese group (*b* = 22.66 _(95% CI = 1.26–44.05)_, t = 2.14, *p* = 0.04) (Table 5; Figure 2).

### 3.6. Regression Analyses

**Figure 3:**
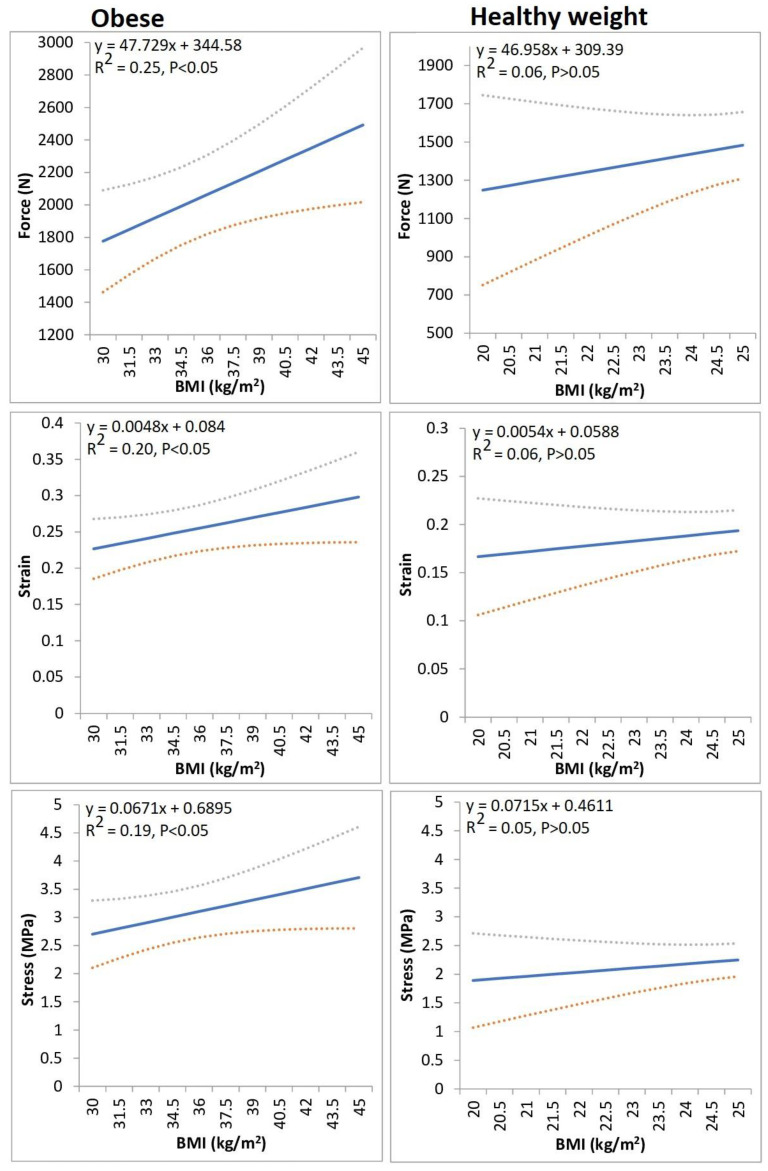
The relationship between BMI and indices of medial tibiofemoral loading. The solid line denotes the linear regression, and the dashed lines characterize its 95% confidence intervals.

In obese individuals BMI significantly predicted peak net medial tibiofemoral joint force, peak stress and peak strain. The regression models showed that peak forces, stress and strains at the medial tibiofemoral compartment were augmented by 47.73 N, 0.005 and 0.07 MPa, respectively, for every 1 unit increase in BMI (Figure 3).

## 4. Discussion

The current study aimed to explore the effects of obesity on medial tibiofemoral cartilage mechanics and longitudinal failure probability, in comparison to healthy individuals using both musculoskeletal simulation and computational modelling approaches. This represents the first investigation to examine the influence of obesity using the aforementioned approaches and may thus yield more comprehensive evidence concerning the influence of obesity on medial knee OA risk.

The plausibility of the medial tibiofemoral mechanical outcomes was inspected through comparisons against previously presented in vivo and other biomechanical modelling information. Taking into account the experimental walking velocities, the peak normalized medial tibiofemoral forces in the healthy weight group were analogous to those using both musculoskeletal modelling (2.90 BW, 1.52 m/s [35]) and simulation techniques (2.73 BW, 1.50 m/s [12]) and also to participant K8L (2.59 BW, 1.39 m/s) from in vivo data [65]. Furthermore, values in obese individuals were also similar to the normalized values obtained from musculoskeletal simulation in asymptomatic obese individuals (2.00 BW, 1.35 m/s [34]). Similarly, the strains experienced by the medial tibiofemoral cartilage were similar to those of Miller & Krupenevich, [35] (0.23) at 1.52 m/s. In comparison to the longitudinal medial tibiofemoral cartilage failure indices, the values in the healthy group are similar to those of Miller & Krupenevich, [35] (13.4%), and in line with the epidemiological literature in the general public for medial tibiofemoral OA aged 60 [11]. Furthermore, the cartilage failure probability values in the obese group are in line with incidence rates presented within the literature [13], and the increased risk for medial knee OA in this group compared to the healthy weight are in agreement with published relative risk indices of 3.78 [66].

In agreement with our hypotheses, the observations from this study importantly revealed that peak net medial tibiofemoral joint forces, stresses and strains were significantly greater in obese individuals in comparison to healthy weight participants. As previous analyses [45] have shown the vastus intermedius, vastus lateralis and vastus medialis muscle forces to be the strongest predictors of medial tibiofemoral joint compartment loading during walking, it is probable that the findings in relation to medial tibiofemoral joint mechanics, were arbitrated as a result of the corresponding increases in vasti muscle kinetics. Our observations support those of Harding et al. [34] who showed that medial tibiofemoral forces were greater in obese individuals. Notably our regression models also showed that in obese individuals BMI significantly predicted indices of medial tibiofemoral joint loading. It is interesting to note that BMI was significantly associated with medial tibiofemoral loading only in obese individuals and not in those who are a healthy weight. Previous analyses have shown significant associations between BMI and medial tibiofemoral strains [67], but this investigation is the first to undertake separate regression models in both obese and healthy weight groups. Therefore, this indicates that there appears to be a threshold above which, BMI appears to have a more pronounced influence on medial tibiofemoral loading indices. Nonetheless, this study does confirm that obesity appears to augment the risk of from the mechanical indices connected to the aetiology of medial knee OA [68].

Notably, in addition to the aforementioned observations concerning medial tibiofemoral loading indices experienced as a function of each footfall, the findings from the current investigation also showed that net medial tibiofemoral cumulative load was also significantly augmented in obese individuals. This observation also supports our hypothesis and allied to the enhanced indices of medial knee joint loading per footfall in the obese group, it would appear that the cumulative joint loads were further exacerbated by the reduced (although not significantly) stride lengths that were found. This importantly meant that a larger number of footfalls with increased medial tibiofemoral loads were required to complete the same modelled distance, which had unequivocal implications for lifetime cartilage failure probability.

Once again in line with our hypotheses, this investigation most importantly revealed, that failure probability was statistically greater in the obese group in comparison to healthy-weight individuals. It is noteworthy that the average failure probability indices of the obese group were 3.99 times greater than in the healthy weight participant group. Taking into account the parameters included in the probabilistic failure model [35], such increases were mediated as a combined function of the significantly greater cartilage strains allied with the increased number of steps required to complete the required daily distance in the obese group. This investigation therefore strongly supports the long-held notion regarding the negative effects of obesity on tibiofemoral cartilage health. This investigation showed BMI to be a significant predictor of cartilage loading indices in obese individuals and previous analyses importantly having revealed that weight loss is able to mediate significant reductions in medial tibiofemoral loading [69,70]. Taking into account the debilitating and painful presentation of knee OA [7] as well as its fiscal healthcare implications [10], the findings from this study therefore strongly advocate for the implementation of effective weight management programs into long-term musculoskeletal management strategies.

Taking into account the modelled walking volume and measured velocity, the current investigation produced similar axial joint forces and cartilage strains to previous musculoskeletal modelling, simulation and in vivo analyses [12,34,35] as well as medial tibiofemoral failure probabilities to epidemiological incidence rates in both healthy weight and obese groups [11,13,35,66]. However, OA is recognized as a multifactorial joint disease [14] in which chronic low-grade inflammation plays an important role [17,18,20,21], therefore as the computational model of medial tibiofemoral cartilage failure probability adopted in the current investigation did not account for adipokine levels, may serve as a limitation to this investigation. Future developmental analyses are necessary to develop a more complex and computationally heavy probabilistic model, capable of quantifying the interaction between mechanical and biochemical mechanisms of medial tibiofemoral OA. A more robust and pathophysiologically relevant probabilistic model of knee OA may allow future disease-modifying therapeutic interventions to be examined more readily. Taking into the debilitating nature of knee OA [10] in addition to its fiscal implications [8], this is an avenue of significant interest for future computational modelling research.

## 5. Conclusions

In conclusion, though walking biomechanics in obese individuals has received considerable research attention, there has not yet been an exploration of the longitudinal effects of obesity using a cumulative musculoskeletal simulation and probabilistic modelling approach. The present study, therefore, enhances current clinical knowledge, by examining the effects of obesity on medial tibiofemoral cartilage failure probability. Importantly, medial tibiofemoral cartilage force, stress and strain were statistically greater in obese individuals in comparison to those of healthy weight. The findings from the current investigation also importantly showed that obesity has a profoundly negative influence on longitudinal knee cartilage health and strongly advocate for the implementation of effective weight management programs into long-term musculoskeletal management strategies.

## Figures and Tables

**Table 1:** Participant characteristic values for the obese and healthy weight groups.

	Healthy Weight				Obese				
	Mean	SD	95% CI Lower	95% CI Upper	Mean	SD	95% CI Lower	95% CI Upper	
Age (yrs)	25.50	4.53	23.49	27.51	24.18	3.19	22.77	25.59	
Mass (kg)	63.18	3.55	61.61	64.76	92.82	5.59	90.34	95.30	*
Stature (m)	1.63	0.05	1.61	1.66	1.63	0.05	1.61	1.66	
BMI (kg/m^2^)	23.71	1.27	23.14	24.27	34.87	2.36	33.82	35.91	*

Notes: * = significant difference between healthy-weight and obese groups.

**Table 2:** Kinematic temporal parameters from normal weight and obese groups.

	Healthy Weight				Obese			
	Mean	SD	95% CI Lower	95% CI Upper	Mean	SD	95% CI Lower	95% CI Upper
Walking velocity (m/s)	1.45	0.24	1.34	1.56	1.38	0.21	1.28	1.48
Stride length (m)	1.61	0.21	1.51	1.70	1.55	0.18	1.46	1.63

**Table 3:** Medial tibiofemoral and muscle forces from normal weight and obese groups.

	Healthy Weight				Obese				
	Mean	SD	95% CI Lower	95% CI Upper	Mean	SD	95% CI Lower	95% CI Upper	
Peak medial tibiofemoral force (BW)	2.22	0.52	1.98	2.46	2.22	0.46	2.01	2.44	
Medial tibiofemoral cumulative load (BW/m)	1.43	0.33	1.28	1.58	1.56	0.32	1.41	1.71	
Net peak medial tibiofemoral force (N)	1493.21	370.19	1319.96	1666.47	2013.92	570.91	1746.72	2281.12	*
Net medial tibiofemoral cumulative load (N/m)	956.11	194.39	865.13	1047.09	1408.30	351.50	1243.79	1572.81	*
Vastus intermedius force (BW)	0.56	0.25	0.44	0.68	0.57	0.19	0.47	0.66	
Vastus lateralis force (BW)	0.49	0.22	0.39	0.60	0.50	0.17	0.42	0.58	
Vastus medialis force (BW)	0.42	0.19	0.33	0.51	0.42	0.15	0.35	0.49	
Net vastus intermedius force (N)	375.85	172.24	295.24	456.47	529.08	272.59	401.50	656.66	*
Net vastus lateralis force (N)	330.71	151.40	259.85	401.56	465.39	239.61	353.25	577.53	*
Net vastus medialis force (N)	280.17	128.23	220.16	340.18	394.00	203.59	298.71	489.28	*

Notes: * = significant difference between healthy-weight and obese groups.

**Table 4:** Medial tibiofemoral contact mechanics from normal weight and obese groups.

	Healthy Weight				Obese				
	Mean	SD	95% CI Lower	95% CI Upper	Mean	SD	95% CI Lower	95% CI Upper	
Peak medial tibiofemoral stress (MPa)	2.26	0.61	1.98	2.55	3.03	1.02	2.56	3.51	*
Peak medial tibiofemoral strain	0.19	0.05	0.17	0.22	0.25	0.07	0.22	0.28	*

Notes: * = significant difference between healthy-weight and obese groups.

**Table 5:** Medial tibiofemoral cartilage failure probabilistic parameters from normal weight and obese groups.

	Healthy Weight				Obese				
	Mean	SD	95% CI Lower	95% CI Upper	Mean	SD	95% CI Lower	95% CI Upper	
Probability of failure (%)	11.63	30.32	3.11	25.82	42.98	47.61	20.70	65.26	*
Probability of failure with repair (%)	7.58	22.84	2.56	18.26	30.23	41.38	10.87	49.60	*

Notes: * = significant difference between healthy-weight and obese groups.

## Data Availability

Not applicable.

## Supplementary Materials

The following supporting information can be downloaded at: https://www.mdpi.com/article/10.3390/life13020270/s1, Figure S1: Anterior femoral arc in sagittal plane; Figure S2: Femoral cartilage modulus; Figure S3: Covered tibial cartilage modulus; Figure S4: Uncovered tibial cartilage modulus; Figure S5: Meniscus modulus; Figure S6: Unloaded cartilage height; Figure S7: Frontal tibial arc; Figure S8: Poisson’s ratio; Table S1: Reliability and MDC values; Table S2: Walking velocity; Table S3: Input peak medial tibiofemoral forces.
