# Supplementary material for: Effects of Obesity on Medial Tibiofemoral Cartilage Mechanics in Females—An Exploration Using Musculoskeletal Simulation and Probabilistic Cartilage Failure Modelling
Source: Life (Basel). 2023 Jan 18;13(2):270. doi: 10.3390/life13020270 (PMC9964246; doi:10.3390/life13020270)

Supplementary Material (S1)

Reliability and MDC values

Table S1: Reliability and MDC values.

	ICC	MDC
Peak medial tibiofemoral force (BW)	0.951	0.27
Peak medial tibiofemoral stress (MPa)	0.947	0.01
Peak medial tibiofemoral strain	0.945	0.16

Note: MDC = 1.96 x SEM x square root of 2.

Supplementary Material (S2)

Sensitivity analysis

Table S2: Walking velocity

	Mean	SD
Walking Velocity (m/s)	1.62	0.21

Table S3: Input peak medial tibiofemoral forces

	Mean	SD
Peak medial tibiofemoral force walking (BW)	3.25	0.55

Linear line of best fit included to highlight the nature of the data distribution.

Figure S1: Anterior femoral arc in sagittal plane

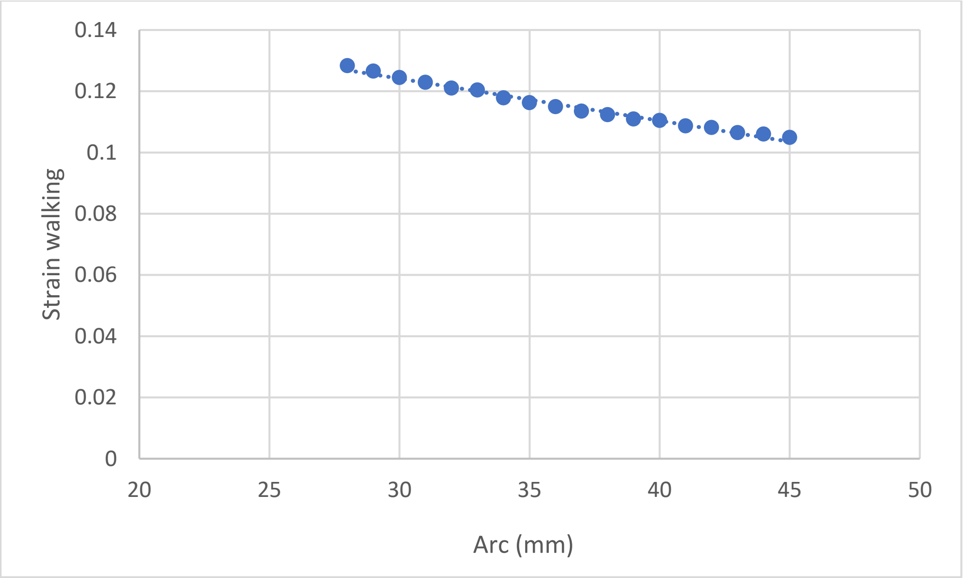

Figure S2: Femoral cartilage modulus

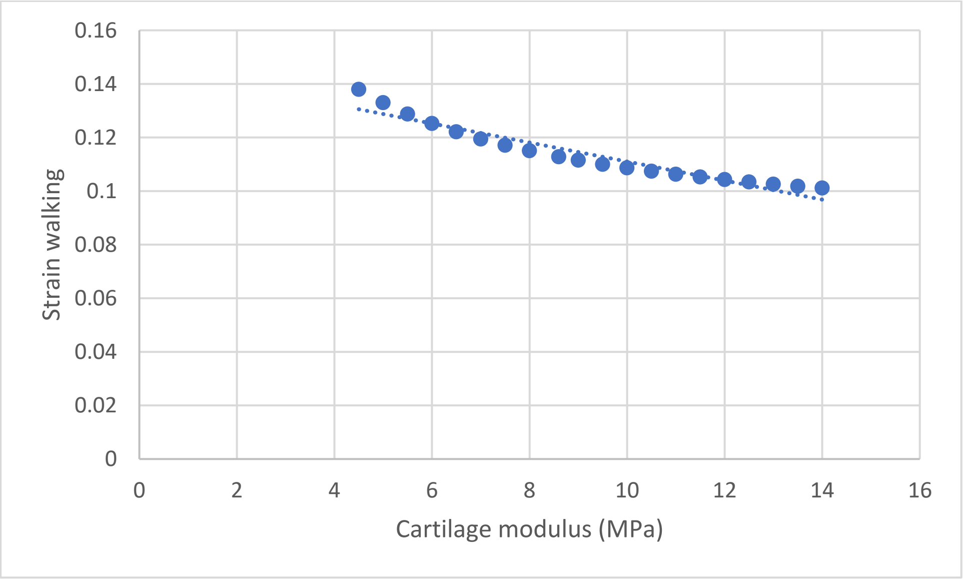

Figure S3: Covered tibial cartilage modulus

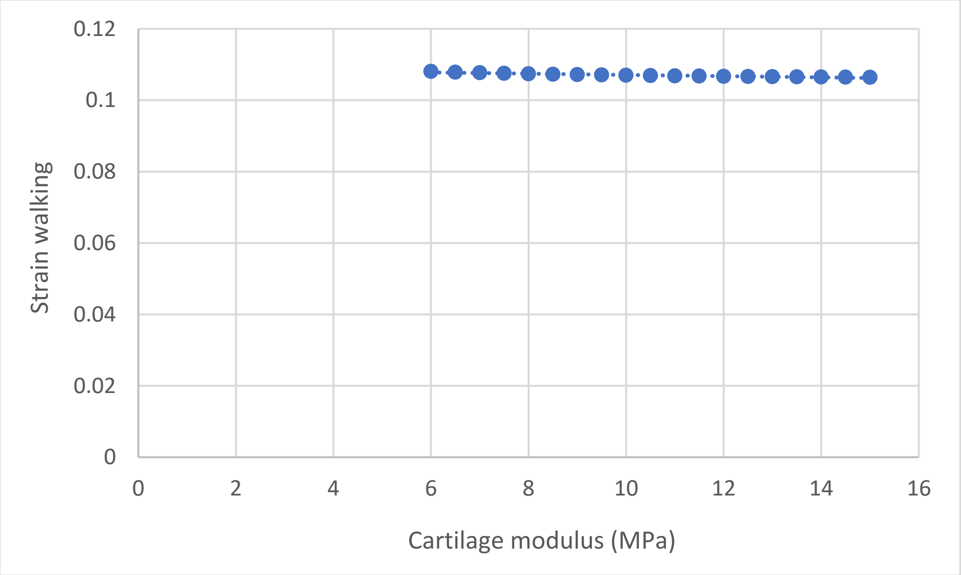

Figure S4: Uncovered tibial cartilage modulus

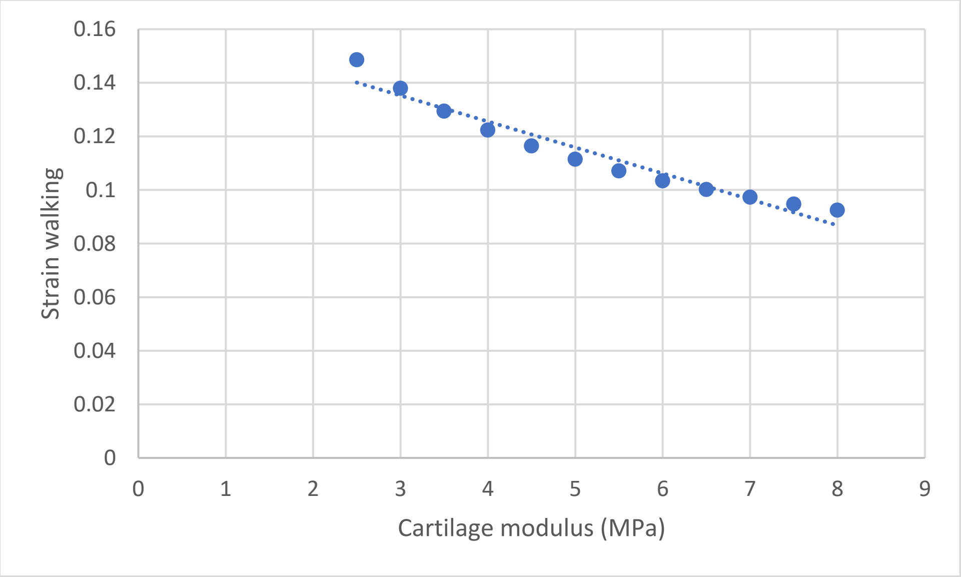

Figure S5: Meniscus modulus

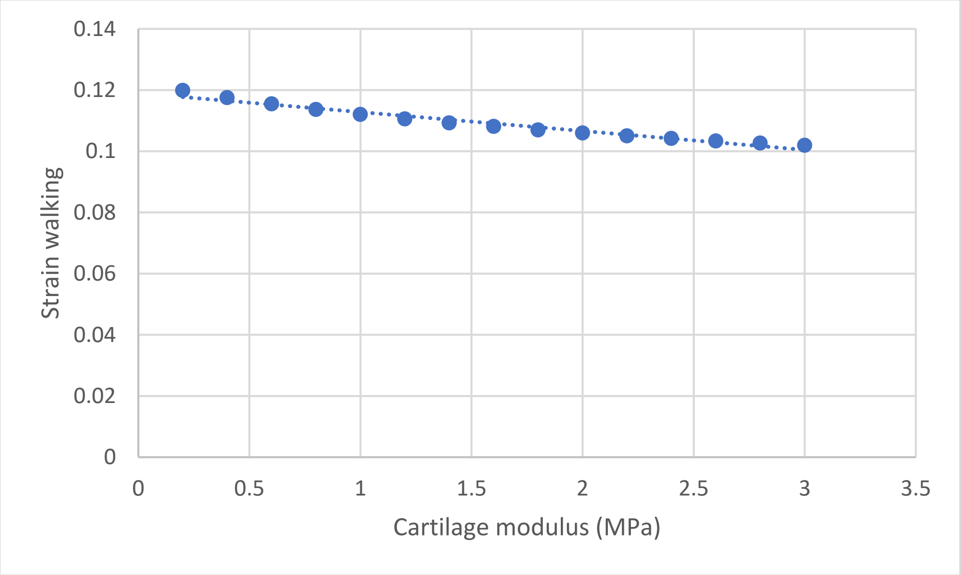

<u>Figure S6: Unloaded cartilage height</u>

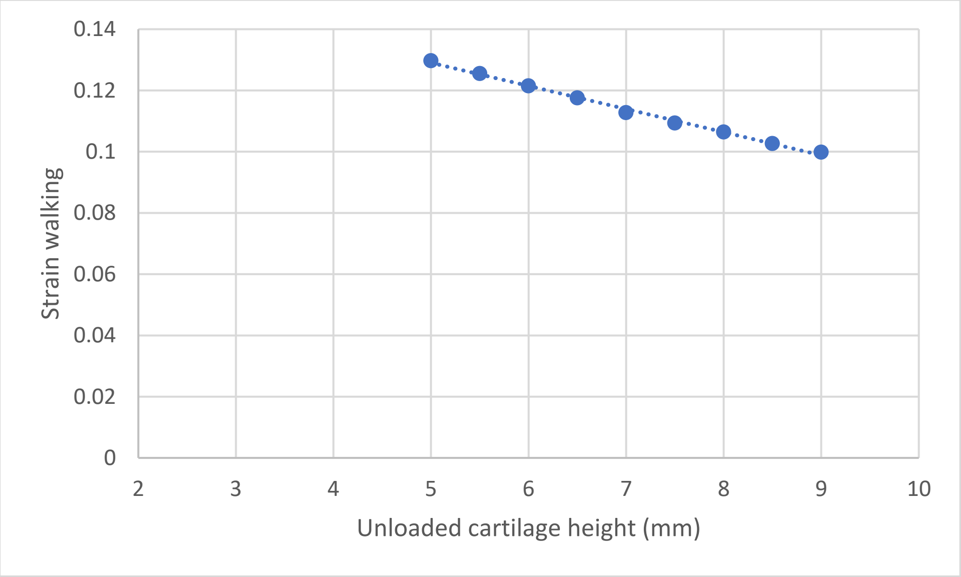

<u>Figure S7: Frontal tibial arc</u>

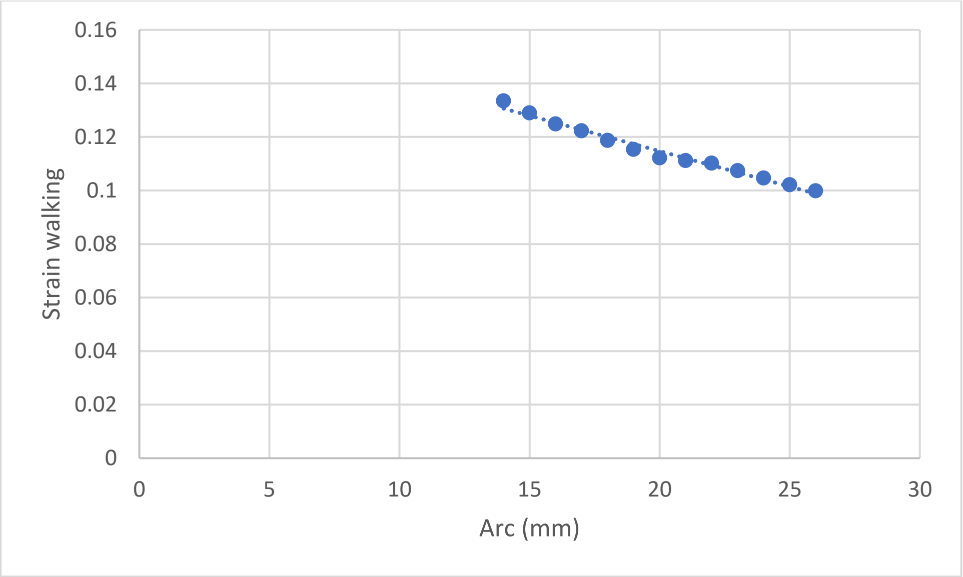

Figure S8: Poisson's ratio

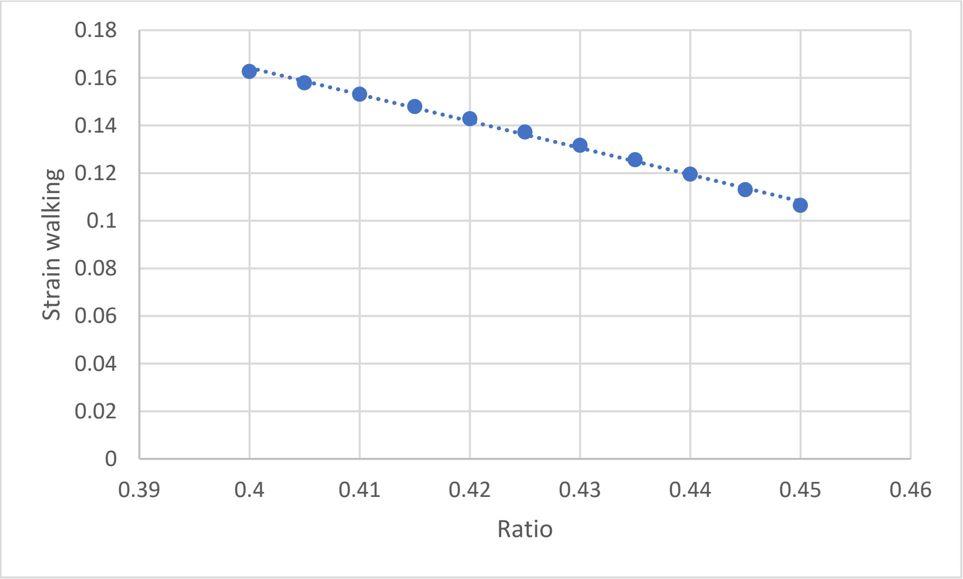

Supplement: Supplementary file 1 [file life-13-00270-s001.zip › life-2053127-supplementary.pdf]
